# Closing oil palm yield gaps among Indonesian smallholders through industry schemes, pruning, weeding and improved seeds

**DOI:** 10.1098/rsos.160292

**Published:** 2016-08-31

**Authors:** T. Soliman, F. K. S. Lim, J. S. H. Lee, L. R. Carrasco

**Affiliations:** 1 The National Institute of Water and Atmospheric Research, 10 Kyle Street, Riccarton, Christchurch 8011, New Zealand; 2 Department of Biological Sciences, National University of Singapore, 14 Science Drive 4, Singapore117543, Republic of Singapore; 3 Asian School of the Environment, Nanyang Technological University of Singapore, 50 Nanyang Avenue, Block N2-01C-37, Singapore639798, Republic of Singapore

**Keywords:** biodiversity, efficiency analysis, Indonesia, land sparing, oil palm, smallholders

## Abstract

Oil palm production has led to large losses of valuable habitats for tropical biodiversity. Sparing of land for nature could in theory be attained if oil palm yields increased. The efficiency of oil palm smallholders is below its potential capacity, but the factors determining efficiency are poorly understood. We employed a two-stage data envelopment analysis approach to assess the influence of agronomic, supply chain and management factors on oil palm production efficiency in 190 smallholders in six villages in Indonesia. The results show that, on average, yield increases of 65% were possible and that fertilizer and herbicide use was excessive and inefficient. Adopting industry-supported scheme management practices, use of high-quality seeds and higher pruning and weeding rates were found to improve efficiency. Smallholder oil palm production intensification in Indonesia has the capacity to increase production by 26%, an equivalent of 1.75 million hectares of land.

## 1. Introduction

Indonesia is the leading producer and exporter of palm oil worldwide, with over 19 million tonnes produced annually [1,2]. In 2008, over 41% of oil palm plantations were owned by smallholders, producing 6.6 million tonnes of palm oil [3]. Most of the increase in Indonesian palm oil production is due to the rapid expansion of plantation area, often at the expense of forests, rather than yield improvements. The expansion of area planted has been estimated in the last two decades to be three million hectares [1]. While absolute area expansion of private estates remains higher, the rate of area expansion among smallholders is increasing faster than among government and private estates [4]. Smallholder yield is estimated to be 50% lower than the potential attainable yield, whereas the yield of government and private estates is estimated to be between 85% and 90% of the potential attainable yield [5].

The continued conversion of primary and logged forests into oil palm plantations has led to significant negative environmental externalities [6]. Decrease in biodiversity, peatland destruction, increased carbon emissions, sedimentation in freshwater systems and air pollution are seen as some of the most prominent negative impacts [7,8]. Agricultural expansion not only poses a threat to biodiversity [7,9,10], but it also contributes to haze events, decreasing air quality across parts of Southeast Asia [11,12].

To avoid the negative environmental impacts of oil palm plantations, while maintaining their productivity, land sparing through agricultural intensification instead of land expansion, is commonly proposed [13]. Land sparing involves intensifying land use for agricultural commodities so as to minimize the area of land needed to meet demand, even though the increased use of some agricultural inputs, such as fertilizers and insecticides, can have negative impacts on biodiversity and freshwater quality when compared with conventional practices [14]. A precondition for any intensification policy to work is to know which factors explain production efficiency. It is, however, not known which production factors, if any, have the potential to increase smallholders' efficiency in Indonesia and whether any managerial, environmental or socio-economic aspects are constraining the efficiency of smallholders' production. Therefore, how much oil palm production improvement is possible and what are the main factors that could increase efficiency, are questions of high importance for biodiversity conservation and sustainability in the tropics. In addition, understanding the underpinning causes of smallholders' inefficiency is pivotal to promote intensification as one of the strategies for conservation policy and for food and financial security among smallholders.

Despite the large potential for production increases without expanding oil palm plantations, the potential improvement in efficiency by Indonesian smallholders has received scant attention. An exception is Fleming & Coelli [15] that assessed the efficiency of a smallholder scheme in West Sumatra as a function of extension advice, gender and education, showing that farmers disseminating extension advice was not effective at improving efficiency. Fleming & Coelli's [15] study further demonstrates how the economic performance of oil palm smallholders can be studied using efficiency measures such as technical, allocative and scale efficiency as well as input and output efficiency.

Here, we aim to estimate the efficiency of smallholder producers in Indonesia while identifying key factors that may indirectly affect the production process. The identification of efficiency factors could help us design appropriate policies for agricultural intensification of smallholder oil palm plantations.

## 2. Methods

The efficiency of smallholder oil palm producers was analysed using a two-stage single-bootstrap data envelopment analysis [16]. In the first stage, an input-oriented data envelopment analysis was conducted, using a set of basic physical variables in order to estimate the efficiency scores of the sampled smallholder producers. The input-oriented technical and scale efficiencies were estimated using the R package ‘Benchmarking’ [17].

In the second stage, a truncated-regression with single bootstrap was conducted in which the estimated efficiency scores were regressed on a set of agronomic, supply chain and managerial factors that might indirectly affect the production process. This procedure was used to approximate the asymptotic distribution of efficiency scores. This method also corrects for the upward bias in estimated efficiency scores that arises from dependency between the first-stage input variables and the second-stage explanatory variables, leading to a correlation between the second-stage explanatory variable and the error term, and from the serial correlation that exists between the estimated efficiency scores [16,18].

### 2.1. Theoretical model

#### 2.1.1. First-stage analysis: input-oriented data envelopment analysis

We assume that a production technology on a sample data *j* of oil palm smallholders is defined by an output vector $y\in {ℜ}_{+}^{M}$ of *M* output dimensions that can be produced by an input vector $x\in {ℜ}_{+}^{N}$ of *N* input dimensions. The technology (*T*) is given by

2.1 T={(x,y) such that x can produce y}.

The technical efficiency (TE) scores are estimated for each *j* producer using the Farrell/Debreu-type input-oriented TE measure [19,20]:

2.2 $$\mathrm{TE}(x^{j},y^{j})=\underset{\theta }{\min}\{\theta :(\theta x^{j},y^{j})\in T\}.$$

As (*T*) is unobserved, we estimate ($\hat{T}$) such that

2.3 $$\hat{T}=\{(x,y)\in {ℜ}_{+}^{N}x{ℜ}_{+}^{M}:\sum_{k=1}^{n}z_{k}y_{m}^{k}\geq y_{m},m=1,\ldots ,M,\sum_{k=1}^{n}z_{k}x_{i}^{k}\leq x_{i},i=1,\ldots ,N,z_{k}\geq 0,k=1,\ldots ,n\},$$

where $\hat{T}$ is an estimator of the unknown true technology set *T* that encloses the production input–output data. *z_k_* is the weight to be used as multiplier for the input levels of a referent producer to indicate the input levels that an inefficient producer should attain in order to achieve efficiency. The TE score lies between zero and one, with one representing the most efficient producers.

For our analysis, we calculated the efficiency scores under four returns to scale assumptions: constant returns to scale (CRS), variable returns to scale (VRS), increasing returns to scale (IRS) and decreasing returns to scale (DRS). To adapt equation (2.3) to these assumptions, we added the condition $\sum_{k=1}^{n}z_{k}\leq 1$ for the DRS assumption, $\sum_{k=1}^{n}z_{k}\geq 1$ for the IRS assumption, and the condition $\sum_{k=1}^{n}z_{k}=1$ for the VRS assumption.

The scale efficiency can then be calculated by

$$\mathrm{SE}=\frac{TE_{\mathrm{CRS}}}{TE_{\mathrm{VRS}}},$$

where TE_CRS_ is the estimated TE scores under the CRS assumption, whereas TE_VRS_ is the estimated TE scores under the VRS assumption.

#### 2.1.2. Second-stage analysis: truncated-regression with single bootstrap

Using maximum-likelihood, the TE scores were regressed on the agronomic, supply chain and managerial factors (*z*_*j*_) as follows [16]

2.4 $$\mathrm{TE}=a+z_{j}\delta +\varepsilon_{j},\;j=1,\ldots ,n,$$

where

2.5 $$\varepsilon_{j}∼N(0,\sigma_{\varepsilon }^{2}),\;\mathrm{such}\;\mathrm{that}\;\varepsilon_{j}\geq 1-a-z_{j}\delta ,j=1,\ldots ,n.$$

The truncated regression ensures that values of the efficiency scores ranged between zero and one. The error term (σ_*j*_) is assumed to be normally distributed with zero mean and unknown variance ($\sigma_{\varepsilon }^{2}$) and a left truncation at 1 − *a* − *z_j_δ*, where *a* and *δ* are the intercept and slope parameters in the regression. We employed a bootstrap approach to increase the precision of the estimation of the confidence intervals.

### 2.2. Data collection and variables

The survey was conducted in two Indonesian regencies where oil palm plantations have been established approximately 30 years ago: Musi Banyuasin, from the province of South Sumatra, and Pasaman Barat, from the province of West Sumatra [21]. Based on reports provided by the Department of Plantation (*Dinas Perkebunan*), smallholder oil palm plantations made up 39% (87 000 ha) and 64% (96 000 ha) of total planted oil palm area in each province, respectively [22]. A farm-level survey was conducted in June and July 2011, using a standardized questionnaire that consisted of two sections: (i) socio-economic background of the smallholder's household and (ii) characteristics of their oil palm plantations. The dataset consists of a stratified random sample survey of 190 producers in six different villages (tables 1 and 2). Maps of main roads and landmarks were used in each village to locate random starting points for transect lines that were followed to choose the households entering the study. The head of the household responded to the survey unless the head was away [21].

**Table 1.** Description of different variables recorded and used for analyses (further details are available in electronic supplementary material, table S1).

variable	description
yield (kg per hectare)	dependent variable used to determine efficiency. Calculated representing the annual yield of fresh fruit bunch from oil palm smallholding
land area (hectares)	the total area of smallholder oil palm plantation
fertilizer usage (kg per hectare)	the annual usage of nitrogen fertilizer per hectare of land
herbicide usage (kg per hectare)	the volume of herbicide applied per hectare and year
seedling quality	seedling quality, based on where smallholders source their seedlings from. The seed variable is categorized as high, medium and low
harvest rate	the number of harvesting rotations of fresh fruit bunches per month
distance to mill (km)	the distance of smallholding to nearest available oil palm mill
distance to road (km)	the distance of smallholding to nearest main tarred road
management practice	the main method of managing the plantations. Categorized either as independent or scheme management
ownership	the way of managing the plantations categorized either as independent, scheme, or mixed
tenure	the type of land tenure security
pruning	frequency of pruning of palm fronds in the smallholding
weeding frequency	the frequency of weeding within the oil palm smallholding
soil type	the main soil type within the smallholding

**Table 2.** Summary statistics for data on oil palm smallholder producers in Indonesia. The mean, interquartile range and median are given for continuous variables. The frequency for each level is given for categorical variables.

variable	mean	s.d.	minimum	first quartile	median	third quartile	maximum
yield	15 634	6561	600	12 000	15 633	21 000	32 256
land	1.73	1.22	0.125	1	2	2	10
fertilizer	134	147.5	0	37.88	112.7	161	920
herbicide	2.23	2.08	0	0.69	2	3.45	10
mill	7.82	3.27	4	7	7	7	17
road	0.17	0.43	0	0	0	0.1	3
**variable**	**categories**						**frequency**
management	independent; scheme						132; 58
ownership	independent; mixed; scheme						98; 35; 57
soil	mineral; swamp						166; 24
seed	low; medium; high; mixed						13; 50; 125; 2
tenure	minimum; moderate; maximum						13; 21; 156
harvest rate	none; once; twice						22; 10; 158
pruning	none; every three months; every six months; every year; once; other						24; 14; 87; 17; 29; 19
weeding	none; every three months; every four months; every six months; every year; other						43; 26; 10; 72; 12; 27

#### 2.2.1. First-stage data analysis: calculating the efficiency of oil palm producers

To calculate the efficiency of oil palm production, we relied on results from the survey, in particular, questions regarding yield, land area and usage of fertilizers and herbicides (table 1, dataset is available in electronic supplementary material, table S1).

#### 2.2.2. Second-stage data analysis: identifying managerial factors that influence efficiency

The survey also focused on different aspects of running the plantation such as (i) number of harvest rotations per month; (ii) distance of the nearest road and oil palm mill from each plantation, as it may be important in reducing transport costs; (iii) weeding and pruning frequencies, because both are common practices that may improve yield; (iv) age of oil palm trees as tree age is known to have an effect on yield; (v) soil type; and (vi) whether the land was waterlogged or not (tables 1 and 2).

Another set of variables recorded was the major form of management practice and ownership. Smallholdings managed almost entirely by smallholders, without agronomic inputs or technical assistance from companies, were considered independent. In contrast, smallholders with some level of input provided by companies were considered to follow ‘scheme management’. In addition, we noted the type of ownership each smallholder had; a smallholder could own only independent smallholdings (classified as independent), only scheme holdings (scheme) or a mixture of both (mixed) independent and scheme smallholdings. We also collected information about the type of land tenure security they had. Smallholders may have minimum tenure security (only verbal agreements or by customary law), moderate land tenure security (where land titles such as *Surat Keterangan Tanah*, *Sopradik* and *Segel* are issued from the village or regency), or maximum land tenure security (where land titles such as the *Badan Pertanahan Nasional* land certificate have been granted by the National Land Planning Agency).

Continuous variables were standardized, so results of the regression were directly comparable. We ran a simple linear regression and checked for collinearity among variables using the *vif* function in the ‘car’ package [23]. Management practice and smallholder ownership were highly collinear, and therefore, we removed ownership type. We then ran the truncated regression with all remaining explanatory variables, and bootstrapped the truncated regression across 1000 iterations. These analyses were conducted using the *treg* function found in the ‘FEAR’ package [24] in R [25].

## 3. Results

### 3.1. First-stage data analysis: efficiency of production

**Figure 1.**
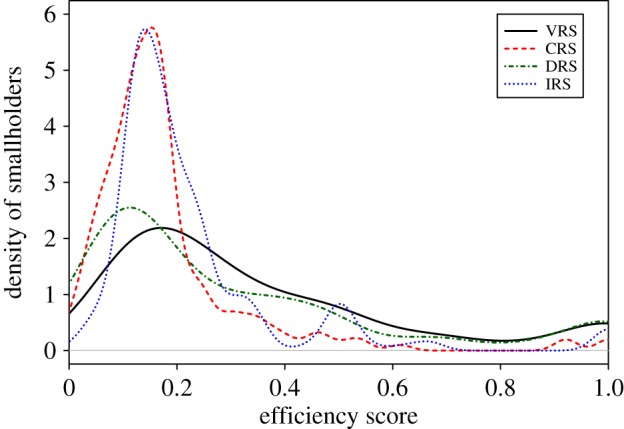
Density plot shows the distribution of efficiency scores across all smallholders surveyed under constant returns to scale (CRS), variable returns to scale (VRS), increasing returns to scale (IRS), and decreasing returns to scale (DRS) assumptions.

Assuming VRS, the results showed that 10% (or 19) of the smallholders were operating at the frontier (efficiency score = 1), whereas 56% presented an efficiency score below 0.3 (figure 1). The mean and median of the efficiency scores were estimated at 0.35 and 0.24, respectively, indicating a high potential for improving TE (table 3 and figure 1). For instance, oil palm smallholders, on average, could reduce their herbicide and fertilizer use by 65% and maintain the same yield. The mean of the efficiency scores under VRS (0.35) was higher than the mean of the efficiency scores under decreasing returns to scale DRS (0.3), increasing returns to scale IRS (0.22) and constant returns to scale CRS (0.17; figure 1 and table 3). This is because VRS allows for the largest possible technology set compared with DRS, IRS and CRS. The DRS enlarges the technology set for small input values, whereas the IRS enlarges the technology set for the large input values. Regarding the scale efficiency, the mean and median scores were estimated at 0.58 and 0.56, respectively, with a standard deviation of 0.27. In addition, 95 smallholders were estimated to produce at a scale below the optimal scale size.

**Table 3.** First-stage input-oriented efficiency results. RS, returns to scale. Constant returns to scale (CRS), variable returns to scale (VRS), increasing returns to scale (IRS), and decreasing returns to scale (DRS).

efficiency	RS	mean	s.d.	first quartile	median	third quartile
technical	VRS	0.35	0.27	0.12	0.24	0.48
technical	CRS	0.17	0.15	0.1	0.14	0.19
technical	DRS	0.3	0.28	0.1	0.17	0.4
technical	IRS	0.22	0.17	0.12	0.17	0.24
scale	—	0.58	0.27	0.35	0.56	0.82

### 3.2. Second-stage data analysis: correlates of efficiency

**Figure 2.**
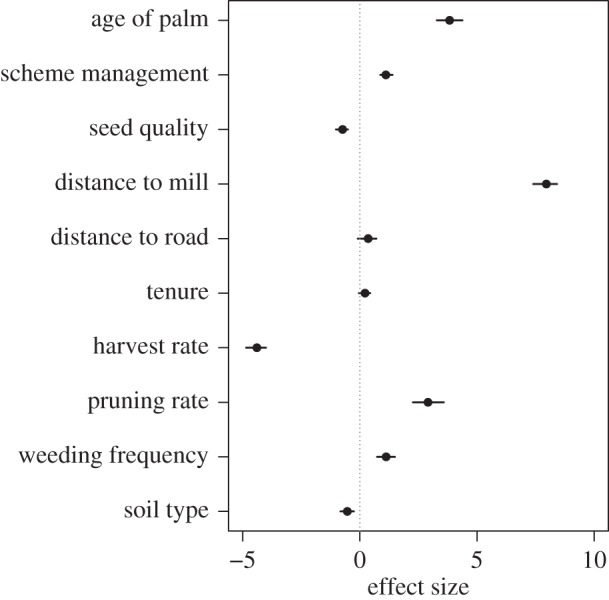
Graph shows effect sizes of each variable from multiple iterations of the truncated regression. Black dots show median effect sizes over 1000 iterations. Error bars denote the 95% range of effect sizes across the 1000 iterations. Scheme management refers to smallholders managed with the support of an oil palm company versus independent smallholders. Soil type corresponds to swampy or waterlogged versus mineral and mixed soils. Tenure corresponds to maximum tenure versus minimum and moderate tenure. Seed quality refers to level ‘low quality’ versus ‘medium’ and ‘high qualities’ (electronic supplementary material, table S1).

Results from the bootstrapped truncated regression suggested that distance from oil palm mill, the age of oil palm and the number of harvest rotations were influential factors on efficiency (figure 2). Scheme management practice had a positive effect (1.11) on efficiency scores: smallholders who adopted scheme management practices were more efficient. Pruning and weeding rates also had a positive effect on efficiency. Seed quality and soil type both showed considerable negative effects: low quality of seeds and smallholdings on swampy or waterlogged soils presented lower efficiency scores (figure 2).

Distance from the mill showed the strongest positive effect on efficiency (median effect size = 7.95). This suggests that plantations farther from the mill were more efficient. The age of oil palm had a strong positive effect (3.83) on production efficiency, i.e. plantations with older palms had higher efficiency. The number of harvest rotations, however, showed a negative effect on production efficiency (−4.39, figure 2).

## 4. Discussion

Only a small percentage of the farmers surveyed were operating at the frontier of production. At least half of the 190 smallholders surveyed received an efficiency score of 0.3 or less. Conservatively using VRS, the average efficiency was 35%, showing a large potential (65%) for yield increases by smallholders.

Scheme smallholders that received some form of input or help from large companies were more efficient. Most of the smallholders surveyed operated independently: only 58 adopted a scheme management practice. Across Indonesia, however, it is estimated that there are about equal numbers of scheme and independent smallholders [26]. By joining an industry-supported scheme, a smallholder can have access to better technology and farming infrastructure, and subsequently achieve yield improvement. However, the smallholders' decision to join a scheme is dependent on whether the additional gains from increasing their productivity exceeds the costs of joining the scheme. Companies do not impose any additional charges for smallholders to join their schemes; however, they deduct all capital costs of land-clearing, seedlings and fertilizers from the sale of the smallholder's fresh fruits to the mill after the oil palm matures.

Age of oil palm also showed a positive effect on efficiency: plantations with older palms presented higher yields. We noted, however, that across all the cases in our study, oil palms were below 20 years of age. These results are thus expected, because yield increases with age up to 14–15 years old and then plateaus until 19–20 years to later decline [27]. The effect of age could also indicate that only the most efficient plantations are maintained until the trees are old; inefficient plantations would have already been abandoned. This would further indicate that our efficiency estimates are conservative, because abandoned plantations could not be included in the study.

Efficiency among smallholders also increased with pruning and weeding frequencies. Management practices such as these may be tied closely with availability of labour. Scarcity of labour is one of the main constraints to oil palm production, and this may be higher among smallholders [28]. These results contrast to those by Prescott *et al*. [29] showing that epiphytes do not reduce yield, and therefore should be left uncut to enhance the habitat for biodiversity conservation. These contradictions highlight the importance of distinguishing between specific types of plants from a farm management perspective.

Efficiency was negatively associated with the number of harvest rotations; among the smallholders interviewed, most of them had harvest rates of two harvests within a month. This is a seemingly counter-intuitive result as the capacity to harvest oil palm kernels when ripe increases the oil quality and extraction ratio. This result could, however, be explained by smallholdings composed of heterogeneous trees from different seeds, thus ripening at different times and requiring more labour inputs at harvest. Synchronized ripening is indeed one of the aims of seed improvement to increase harvesting efficiency [30]. Thus, this result may also be related to the finding that low seed quality reduces efficiency and the typical low reliability of oil palm seed supply among smallholders [31].

As expected, efficiency was lower in waterlogged or swampy soils. This finding has environmental implications as it shows that, given the available choice between mineral land and peat swamp forests, yields are likely to be higher in mineral soil. Adding up the large CO_2_ emissions and impacts on biodiversity as a result of peat swamp conversion to oil palm (although species diversity is known to be lower in peat swamps compared with mineral soils [32]), this result strengthens the case of avoiding conversion of peat swamps into oil palm plantations [33,34].

Contrary to what was expected, short distance to a mill was associated with less efficient smallholders. We note, however, that the distance to the nearest mill may be of only relative importance if scheme smallholders are awarded better access to mills over independent smallholders. In these cases, large companies often ensure smooth transport of fruits to mills, making participation of the scheme more influential than distance itself. Another explanation could be that distance to road is serving as an instrumental variable that represents the probability of a farmer taking part in industry schemes. Distance of plantations to roads had a small effect on efficiency. Accessibility, or distance to tarred roads, varied only slightly across all the surveyed participants: all the plantations were within 3 km of the nearest tarred road, with most being next to the road.

Assuming that the smallholder producers could attain their maximum possible efficiency, their production could increase from 6.6 to 18.9 million tonnes (i.e. an increase in efficiency from 35% to 100%). Given that 41% of the total plantation area across Indonesia is run by smallholders, this increase in production would be equivalent to 12.3 million tonnes and around 1.75 million hectares of land (i.e. 26% of the total land of cultivated oil palm in Indonesia), assuming that the average production is 7 tonnes per hectare. This means that through intensification of smallholders' production, demand could be met without further forest expansion of up to 1.75 million hectares. It should be noted, however, that these estimates are predicated on the assumption that smallholders contribute to forest conversion together with companies. This seems plausible given that smallholders in Sumatra were behind 10.7% of oil palm-driven forest conversion and show higher annual rates of expansion than private companies [35].

Agricultural intensification, especially in the form of herbicide and fertilizer increased use, is, however, known to affect biotic interactions and resource availability in ecosystems, leading to persistent negative effects on biodiversity [36,37]. In this respect, one key result was that smallholders could reduce their herbicide and fertilizer use substantially and maintain the same yield. This indicates a potential win–win situation between production and biodiversity conservation with smallholder intensification policies in Indonesia: better management of herbicides and fertilizer can reduce costs and their concomitant environmental impacts. In this respect, better seeds, joining industry schemes and better pruning techniques represent changes that do not necessarily need to be at odds with biodiversity.

With the rapid increase in global demand for oil palm, it is expected that deforestation will accelerate to make way for expansion of plantations [38]. Oil palm production among smallholders is typically seen as inefficient, in comparison with large companies. Improving smallholders' efficiency could reduce this gap, and increase the equity of the distribution of benefits from oil palm beyond large multinational agri-businesses. Improving yield and efficiency of smallholdings, thereby could in theory spare more land through meeting demand, thus having less negative impact on the environment than low-yield and inefficient plantation expansion [39,40].

Our analyses do not consider, however, the long-term secondary or spill-over effects of improving smallholders' efficiency on agricultural expansion. These may include migration of labour, changes in economic decisions and management practices among smallholders. Increased yields would alter land rent and may even increase capital and incentivize smallholders to expand their plantations further into forests [41]. Clear tenure regimes and governance would thus be needed to prevent undesired feedback effects in the form of further forest encroachment [42]. Other strategies to avoid rebound effects are area zonation, spatial strategic deployment of technology or certification [43]. In this respect, our analyses allow identifying the factors that could increase production efficiency, thus setting the necessary information to support intensification policies. It should, however, be noted that it is beyond the scope of the study to predict the effects of these intensification policies on land-use dynamics, which should be a matter of further research.

Besides studying the land-use implications of closing yield gaps among Indonesian smallholders in six villages, future research could attempt to widen the scope of the analysis to smallholders and oil palm companies across the whole of Indonesia. An integrated network of data collection and monitoring on inputs and outputs in oil palm production could help improve real-time management practices via similar analytical methods to those we used here. The inclusion of biodiversity levels in plantations as an output together with yields could also help identify practices that both lead to production and environmental benefits [44]. In addition, further research could also investigate the factors affecting the probability of a farmer joining industry schemes and ways to estimate the entrepreneurship capacity of different smallholders.

## 5. Conclusion

Meeting the world's demand for oil palm may be achieved by improving yield of existing oil palm agricultural land, as opposed to expansion of plantations. Our analyses show that a large proportion of the Indonesian smallholders surveyed have been operating at low efficiency, with a large potential for fertilizer and herbicide input reduction. This study points out possible ways of altering management strategies to improve efficiency. Future intensification policies should be aimed at higher integration of smallholders into scheme management programmes with large companies, training to adjust management practices involving weeding, pruning, fertilizer and herbicide application and distribution of high-quality seeds to smallholders. Our results support simple policies that could reduce deforestation without compromising palm oil production to meet growing global demands.

## Supplementary Material

Table S1. Raw data

## Data accessibility

The data used for the analyses are available in the electronic supplementary material.

## References

[1] FAO. 2015 FAOSTAT Online Statistical Service (cited 2015). See http://faostat.fao.org/.

[2] World Bank. 2011 The World Bank Group framework and IFC strategy for engagement in the palm oil sector. Washington, DC: International Finance Corporation.

[3] Mahmud A, Rehrig M, Hills G 2010 Improving the livelihoods of palm oil smallholders: the role of the private sector. FSG report. Accessed from: http://www.fsg.org/publications/improving-livelihoods-palm-oil-smallholders#download-area.

[4] Indonesian Palm Oil Council. 2010 Indonesian palm oil in numbers 2010. Jakarta, Indonesia: Indonesian Palm Oil Producers Association.

[5] Molenaar JW, Orth M, Lord S, Meekers P, Taylor C, Hanu MDA, Elson D, Ginting L 2010 Analysis of the agronomic and institutional constraints to smallholder yield improvement in Indonesia. Aidenvironment, Global Sustainability Associates. See http://www.aidenvironment.org/media/uploads/documents/201309_IFC2013_Diagnostic_Study_on_Indonesian_Palm_Oil_Smallholders.pdf.

[6] Fitzherbert EB *et al* 2008 How will oil palm expansion affect biodiversity? Trends Ecol. Evol. 2, 538–545. 10.1016/j.tree.2008.06.012 18775582

[7] Sodhi NS, Koh LP, Brook BW, Ng PK 2004 Southeast Asian biodiversity: an impending disaster. Trends Ecol. Evol. 19, 654–660. (doi:10.1016/j.tree.2004.09.006) 16701328 10.1016/j.tree.2004.09.006

[8] Wilcove DS, Giam X, Edwards DP, Fisher B, Koh LP 2013 Navjot's nightmare revisited: logging, agriculture, and biodiversity in Southeast Asia. Trends Ecol. Evol. 28, 531–540. (doi:10.1016/j.tree.2013.04.005) 23764258 10.1016/j.tree.2013.04.005

[9] Brook BW, Bradshaw CJ, Koh LP, Sodhi NS 2006 Momentum drives the crash: mass extinction in the tropics. Biotropica 38, 302–305. (doi:10.1111/j.1744-7429.2006.00141.x)

[10] Brook BW, Sodhi NS, Ng PK 2003 Catastrophic extinctions follow deforestation in Singapore. Nature 424, 420–426. (doi:10.1038/nature01795) 12879068 10.1038/nature01795

[11] Lee JSH, Jaafar Z, Tan AKJ, Carrasco LR, Ewing JJ, Bickford DP, Webb EL, Koh LP 2016 Toward clearer skies: challenges in regulating transboundary haze in Southeast Asia. Environ. Sci. Policy 55, 87–95. (doi:10.1016/j.envsci.2015.09.008)

[12] Gaveau DL et al. 2014 Major atmospheric emissions from peat fires in Southeast Asia during non-drought years: evidence from the 2013 Sumatran fires. Sci. Rep. 4, 6112 (doi:10.1038/srep06112) 25135165 10.1038/srep06112 PMC4137341

[13] Phalan B, Onial M, Balmford A, Green RE 2011 Reconciling food production and biodiversity conservation: land sharing and land sparing compared. Science 333, 1289–1291. (doi:10.1126/science.1208742) 21885781 10.1126/science.1208742

[14] Firbank LG 2005 Striking a new balance between agricultural production and biodiversity. Ann. Appl. Biol. 146, 163–175. (doi:10.1111/j.1744-7348.2005.040078.x)

[15] Fleming E, Coelli T 2004 Assessing the performance of a nucleus estate and smallholder scheme for oil palm production in West Sumatra: a stochastic frontier analysis. Agric. Syst. 79, 17–30. (doi:10.1016/S0308-521X(03)00043-X)

[16] Simar L, Wilson PW 2007 Estimation and inference in two-stage, semi-parametric models of production processes. J. Economet. 136, 31–64. (doi:10.1016/j.jeconom.2005.07.009)

[17] Bogetoft P, Otto L 2010 Benchmarking with DEA, SFA, and R. Berlin, Germany: Springer Science & Business Media.

[18] Barros CP, Dieke PU 2008 Measuring the economic efficiency of airports: a Simar–Wilson methodology analysis. Transp. Res. E: Logist. Transp. Rev. 44, 1039–1051. (doi:10.1016/j.tre.2008.01.001)

[19] Coelli TJ, Rao DSP, O'Donnell CJ, Battese GE 2005 An introduction to efficiency and productivity analysis. Berlin, Germany: Springer Science & Business Media.

[20] Farrell MJ 1957 The measurement of productive efficiency. J. R. Stat. Soc. A (Gen.) 120, 253–290. (doi:10.2307/2343100)

[21] Lee JSH, Ghazoul J, Obidzinski K, Koh LP 2014 Oil palm smallholder yields and incomes constrained by harvesting practices and type of smallholder management in Indonesia. Agron. Sustain. Dev. 34, 501–513. (doi:10.1007/s13593-013-0159-4)

[22] Indonesian Ministry of Agriculture. 2011 Tree crop estate statistics of Indonesia 2010–2012 oil palm. Jakarta, Indonesia: Indonesian Ministry of Agriculture.

[23] Fox J, Weisberg S 2011 An R companion to applied regression. Thousand Oaks, CA: Sage.

[24] Wilson PW 2008 FEAR: a software package for frontier efficiency analysis with R. Socio-Econ. Plann. Sci. 42, 247–254. (doi:10.1016/j.seps.2007.02.001)

[25] R Development Core Team. 2013 R: a language and environment for statistical computing. Vienna, Austria: R Foundation for Statistical Computing http://www.R-project.org R Foundation for Statistical Computing, Vienna, Austria.

[26] Colchester M 2011 Oil palm expansion in South East Asia: trends and implications for local communities and indigenous peoples. Moreton-in-Marsh, UK: Forest Peoples Programme.

[27] USDA. 2012 Malaysia: stagnating palm oil yields impede growth. United States Department of Agriculture. Foreign Agricultural Service. Accessed at: http://www.pecad.fas.usda.gov/highlights/2012/12/Malaysia/.

[28] Carrasco L, Larrosa C, Milner-Gulland E, Edwards D 2014 Tropical crops: cautious optimism–response. Science 346, 928–928 (doi:10.1126/science.346.6212.928-b) 10.1126/science.346.6212.928-b 25414295

[29] Prescott GW, Edwards DP, Foster WA 2015 Retaining biodiversity in intensive farmland: epiphyte removal in oil palm plantations does not affect yield. Ecol. Evol. 5, 1944–1954. (doi:10.1002/ece3.1462) 26045947 10.1002/ece3.1462 PMC4449750

[30] Morcillo F et al. 2013 Improving palm oil quality through identification and mapping of the lipase gene causing oil deterioration. Nat. Commun. 4, 2160 (doi:10.1038/ncomms3160) 23857501 10.1038/ncomms3160 PMC3717496

[31] Akpo E, Crane TA, Stomph T-J, Tossou RC, Kossou DK, Vissoh PV, Struik PC 2014 Social institutional dynamics of seed system reliability: the case of oil palm in Benin. Int. J. Agric. Sustain. 12, 214–232. (doi:10.1080/14735903.2014.909634)

[32] Posa MRC, Wijedasa LS, Corlett RT 2011 Biodiversity and conservation of tropical peat swamp forests. Bioscience 61, 49–57. (doi:10.1525/bio.2011.61.1.10)

[33] Koh LP, Miettinen J, Liew SC, Ghazoul J 2011 Remotely sensed evidence of tropical peatland conversion to oil palm. Proc. Natl Acad. Sci. USA 108, 5127–5132. (doi:10.1073/pnas.1018776108) 21383161 10.1073/pnas.1018776108 PMC3064377

[34] Murdiyarso D, Hergoualc'h K, Verchot L 2010 Opportunities for reducing greenhouse gas emissions in tropical peatlands. Proc. Natl Acad. Sci. USA 107, 19 655–19 660. (doi:10.1073/pnas.0911966107) 10.1073/pnas.0911966107 PMC2993379 21081702

[35] Lee JSH, Abood S, Ghazoul J, Barus B, Obidzinski K, Koh LP 2014 Environmental impacts of large-scale oil palm enterprises exceed that of smallholdings in Indonesia. Conserv. Lett. 7, 25–33. (doi:10.1111/conl.12039)

[36] Matson PA, Parton WJ, Power A, Swift M 1997 Agricultural intensification and ecosystem properties. Science 277, 504–509. (doi:10.1126/science.277.5325.504) 20662149 10.1126/science.277.5325.504

[37] Geiger F et al. 2010 Persistent negative effects of pesticides on biodiversity and biological control potential on European farmland. Basic Appl. Ecol. 11, 97–105. (doi:10.1016/j.baae.2009.12.001)

[38] Corley RHV 2009 How much palm oil do we need?. Environ. Sci. Policy 12, 134–139. (doi:10.1016/j.envsci.2008.10.011)

[39] Lee JSH, Garcia-Ulloa J, Ghazoul J, Obidzinski K, Koh LP 2014 Modelling environmental and socio-economic trade-offs associated with land-sparing and land-sharing approaches to oil palm expansion. J. Appl. Ecol. 51, 1366–1377. (doi:10.1111/1365-2664.12286)

[40] Azhar B, Lindenmayer DB, Wood J, Fischer J, Manning A, McElhinny C, Zakaria M 2011 The conservation value of oil palm plantation estates, smallholdings and logged peat swamp forest for birds. Forest Ecol. Manage. 262, 2306–2315. (doi:10.1016/j.foreco.2011.08.026)

[41] Angelsen A 1999 Agricultural expansion and deforestation: modelling the impact of population, market forces and property rights. J. Dev. Econ. 58, 185–218. (doi:10.1016/S0304-3878(98)00108-4)

[42] Phelps J, Carrasco LR, Webb EL, Koh LP, Pascual U 2013 Agricultural intensification escalates future conservation costs. Proc. Natl Acad. Sci. USA 110, 7601–7606. (doi:10.1073/pnas.1220070110) 23589860 10.1073/pnas.1220070110 PMC3651457

[43] Phalan B et al. 2016 How can higher-yield farming help to spare nature? Science 351, 450–451. (doi:10.1126/science.aad0055) 26823413 10.1126/science.aad0055

[44] Areal FJ, Tiffin R, Balcombe KG 2012 Provision of environmental output within a multi-output distance function approach. Ecol. Econ. 78, 47–54. (doi:10.1016/j.ecolecon.2012.03.011)

